# Consumption of dietary folate estimates and its implication for reproductive outcome among women of reproductive age in Kersa: cross-sectional survey

**DOI:** 10.1186/s40795-021-00476-6

**Published:** 2021-11-15

**Authors:** Nega Assefa, Yasir Y. Abdullahi, Aklilu Abraham, Elena C. Hemler, Isabel Madzorera, Yadeta Dessie, Kedir Teji Roba, Wafaie W. Fawzi

**Affiliations:** 1 College of Health and Medical Sciences, Haramaya University, Harar, Ethiopia; 2 Jugal Hospital Department of Obstetrics and Gynecology, Harar, Ethiopia; 3 Department of Global Health and Population, Harvard T.H. Chan School of Public Health, Harvard University, Boston, MA USA; 4 Department of Nutrition, Harvard T.H. Chan School of Public Health, Harvard University, Boston, MA USA; 5 Department of Epidemiology, Harvard T.H. Chan School of Public Health, Harvard University, Boston, MA USA

**Keywords:** Folate consumption, Dietary folate, Food diversity, Daily folate consumption

## Abstract

**Background:**

Dietary folate inadequacy is one the most common micronutrient deficiencies that cause neural tube defect (NTD) among infants in Sub-Saharan African countries. This study aims to determine the dietary intake of folate among women of reproductive age (WRA) of Kersa, Eastern Ethiopia.

**Methods:**

A cross-sectional study took place among voluntary women that were selected from 1140 random households. Using a validated Food Frequency Questionnaire, participant’s weekly dietary intake history of Ethiopian foods and dietary folate intake was worked out. Statistical analysis was done at a 95% confidence interval. Modified Poisson regression was used to identify factors associated with dietary folate consumption.

**Result:**

The estimated median usual intake of folate was 170 μg/d (IQR: 118.3; 252.2) and about 33% of WRA had low folate intake and 73.9% were at risk for folate inadequacy. From the reported food groups, Beans and Peas, Starchy staples, and Vitamin-A rich dark-green leafy vegetables were the top three ranked foods that contributed much of the dietary folate. The following conditions were statistically related to dietary folate inadequacy; women’s age, being in poor wealth index, low dietary diversity, having seasonal employment, and reliance on market food sources.

**Conclusions:**

We found that women’s dietary intake of folate in Kersa is very low and cannot protect their offspring from the risk of having NTD. They could also potentially be predisposed to poor health outcomes. Diversifying and fortification of Ethiopian wheats and salts could decrease the burden of folate deficiency in the country.

## Background

Folate is one of the naturally occurring essential micronutrients found in food [1]. Dietary sources of folate include green leafy vegetables, legumes, egg yolks, liver, and citrus fruits [2]. Folic acid is the synthetic form of the micronutrient and is found in dietary supplements, enriched foods, and pharmaceutical vitamins [3, 4].

Folate deficiency is however a severe public health problem, especially among disadvantaged groups in developing countries [5, 6]. It has been linked to various complications during pregnancy. These include increased risk of maternal anemia, hypertensive disorder, abortion, bleeding, and cardiovascular disease [7, 8]. Folate deficiency has also been commonly cited as a significant risk factor for developing neural tube defects (NTD) in the fetus; affecting more than 300,000 babies worldwide, and 65 infants out of 10,000 births in Ethiopia [9–12].

Folate intakes are often low among women in SSA because access to micronutrient-rich foods and fortified foods is limited, and these foods are expensive, locally unavailable, or unacceptable for cultural or religious reasons [13]. Given that inadequate dietary folate consumption is one of the primary causes of folate deficiency, the World Health Organization (WHO) recommends supplementation with 400 μg of folic acid before pregnancy to decrease the incidence of NTD [14, 15]. Since the development of the central nervous system in the embryo occurs as early as 9 weeks after fertilization, an increment in pre-pregnancy folate levels is the crucial and most appropriate method of reducing NTD and other pregnancy complications due to folate deficiency [16].

Population-wise increment of folate consumption status by fortification of wheat and cereals and availing affordable nutrient-rich food alternatives and eliminating hunger have shown significantly in improving nutritional and the health status of women and their offspring [17, 18]. However, there are no folate fortified foods or enriched food products available in Ethiopia [19].

There is limited information available on the dietary intake of folate among WRA in Ethiopia. This study aims to evaluate dietary folate consumption among WRA in Kersa district of Oromia region, eastern Ethiopia. Further, the study evaluates dietary diversity and other factors associated with folate consumption.

## Methods

### Study design and settings

This study was conducted in the Kersa Health and Demographic Surveillance System (KHDSS) field site in Oromia region in eastern Ethiopia. The HDSS covers 24 kebeles (the lowest administrative unit in Ethiopia) three of which are urban, out of the 38 kebeles in the district. The 2016 national census reported that Kersa had the third-largest in Oromia region with a total population of 350,064, and a population density of 36.8 persons per square kilometers [20].

We conducted a cross-sectional survey among 1200 households in the KHDSS from September to August 2019 [21]. Study participants were selected using proportional allocation to the population size of the study kebeles, followed by random selection of households based on data from the KHDSS database. Eligibility criteria for the study included households with at least one married woman, who was of reproductive age (15–49 years old) and was not pregnant at study recruitment. If more than one woman of reproductive age lived in the household and was present at the time of the interview, a lottery method was used to select one woman for the interview.

### Data collection tool

The participants responded to the questionnaire; which had five sections, including information on socio-demographic characteristics, health information, food choices, and cooking practices, food security, food expenditures, homestead food production, and dietary intake. Data were collected via interviewer-administered tablet-based questionnaires, using an Open Data Kit (ODK) platform.

### Sociodemographic assessment

As the study setting was rural, we classified women’s employment according to the Ethiopian Demographic and health survey definition, as fully-employed, and seasonal and part-time employment [22]. Those who were fully employed had a skilled and stable job working in the 7 days preceding the survey. Hard labor and agricultural employment were categorized as partial and seasonal based on time and experience before the survey. Household wealth was defined using a wealth index, constructed using principal component analysis (PCA) of 10 items describing the household’s asset ownership, housing quality, crowding, and water and sanitation facilities. The wealth index was divided into population tertiles (poor, middle, and rich) [23].

### Anthropometric assessment

Height and weight of WRA were measured in the nearest centimeters (cm) and kilograms (kg) using a stadiometer and standard clinical scale [24].. Body mass index (BMI) was computed as weight in kilograms divided by height in meters squared. Based on BMI, individuals were classified using standard cutoffs as underweight (< 18.5 kg/m^2^), normal weight (18.5–24.9 kg/m^2^), or overweight/obese (≥ 25 kg/m^2^). Overweight was classified as BMI 25–29.9 kg/m^2,^ and obesity BMI ≥30 kg/m^2^ [25].

### Dietary assessment

The outcome of interest was women’s dietary folate intake. Women’s diets were assessed using a non-quantitative food frequency questionnaire (FFQ), locally adapted from a semi-quantitative FFQ validated for use among urban Tanzanian adults [26]. The participants were asked if they consumed 69 different foods items in the past 7 days and the frequency of their consumption in terms of days. The weekly reported consumption of the food items was converted into daily consumption by dividing by seven. The FFQ included locally available common foods and an option to specify other foods. Portion size information was not collected in the current study. We used portion sizes for each food item that were adopted from a recent national survey [27].

We assessed women’s dietary diversity using the Minimum Dietary Diversity for Women (MDD-W) indicator [28]. We grouped foods consumed by women into ten non-overlapping food groups. The 10 food groups are 1) starchy staples, 2) pulses 3) nuts and seeds 4) dairy products 5) flesh foods 6) eggs 7) dark green leafy vegetables 8) vitamin-a rich fruits and vegetables 9) other vegetables, and 10) other fruits [29]. Foods made from grains, cereals, roots, and tubers are grouped into starchy staples. Poultry and all meat products were categorized as flesh foods [30].

A participant was scored as consuming a food group if they ate at least one type of food item comprising that food group daily. We summed up the food groups consumed by women into a dietary diversity score (DDS-W, range 0–10). We categorized women as meeting minimum dietary diversity (MDD) if they consumed at least 5 food groups (DDS ≥5) daily. MDD-W serves as a proxy for micronutrient adequacy [31].

We estimated women’s daily folate consumption by multiplying the mean portion size and folate composition for each reported food item with its daily consumption. We summed up women’s total folate intake based on the reported individual foods in the FFQ. The cutoffs for inadequate folate intake were defined as consuming less than the age- and sex-specific Estimated Average Requirement (EAR) of folate intake of WRA < 250 μg/d [32]. We calculated a binary indicator for adequate folate intake (yes/no). We have also divided the total distribution of folate intake into tertiles and categorized them into low, middle, and high folate intake, respectively.

### Data processing and analysis

Data was analyzed using STATA 16. Means and standard deviations (SDs) were used to describe continuous variables and medians and interquartile ranges for variables that were not normally distributed. Counts and percentages were used to describe categorical variables. Data points with more than 50% missing data and with un-usual amounts (outliers) were removed from the analysis. Bivariate analysis using Modified Poisson regression [33, 34] was undertaken to examine the independent predictors of inadequate folate intake (0 = adequate consumption, 1 = inadequate consumption) and Crude Prevalence Ratios (CPR) and 95% Confidence Interval (CI) estimated. Variables that were significant in the univariate analysis (*p* < 0.2) were included to control for confounding for the final model. We computed Adjusted Prevalence Ratio (APR) by incorporating variables that are significant or assumed to be a confounder. The statistical association level was *p* < 0.05 to identify independent variables associated with inadequate folate consumption.

## Result

We analyzed data from 1134 WRA households that participated in the study. Thirty-nine households refused to participate in the study and 27 participants with missing and outlier data were excluded from the analysis. The mean age of women was 31.1 (± 6.2) years and half of the women had never attended school. Most participants were Muslims and housewives. At least 67.5% of WRA worked full time and 56.2% were in the poor wealth index category. The median weight and height of WRA was 51.0 kg (IQR: 48.0; 56.0) and 157.0 cm (IQR: 154.5; 161.1), respectively (Table 1).

**Table 1:** Sociodemographic, reproductive, and Food intake characteristics of women of reproductive age in Kersa, Eastern Ethiopia, 2019

Variables	N	Values
Woman’s age (years)	1134	
16–25		268 (23.6)
26–35		637 (56.2)
≥ 36		229 (20.2)
Highest Education	1134	
Never attended school		609 (50.7)
Did not finished first grade		99 (8.7)
Completed 10 grade and more		426 (37.6)
Partner Highest Education	1134	
Never attended school		571 (50.4)
Did not finished first grade		84 (7.4)
Completed 10 grade and more		479 (42.2)
Religion	1134	
Muslim		1080 (95.2)
Orthodox		42 (3.7)
Other^b^		12 (1.1)
Employment type	1134	
Full-time		765 (67.5)
Part-time		101 (8.9)
seasonal		268 (23.6)
Occupational status of women	1134	
Farmer		925 (81.6)
Trade		49 (4.3)
Professional/technical		66 (5.8)
Other^d^		94 (8.3)
Role in the household	1134	
Head of the HH		141 (12.4)
Spouse		981 (86.5)
Another^c^		12 (1.1)
Wealth index	1134	
Poor		637 (56.2)
Middle		265 (23.4)
Rich		232 (20.4)
Weight^a^ (kg)	1134	51.0 (48.0; 56.0)
Height^a^ (cm)	1134	157.0 (154.5; 161.1)
Body Mass Index (BMI)^a^	1134	20.6 (19.2; 22.4)
BMI		
Underweight	1134	188 (16.6)
Normal		870 (76.7)
Overweight		76 (6.7)
Family size^#^	1134	5.8 ± (3.0)
Has an under 5 children	1134	1029 (90.7)
Age of Under 5 children^a^*	1029	36.0 (23.0; 48.0)
Number of previous pregnancies^#^	1134	4.5 ± (2.4)
Source of household food	1134	
Household production		854 (75.3)
Street Vendor and local market		280 (24.7)
Food source Distance from Household^#e^	280	0.7 ± (1.4)
Women’s Minimum dietary diversity scores (out of 10 groups)^a^	1134	4.0 (3.0; 5.0)
Minimum dietary diversity	1134	
Low		733 (64.6)
Estimated Usual Folate intake (ug/d)^a^	1134	170.2 (118.3; 252.2)
Estimated Folate inadequacy	1134	838 (73.9)
Tertile of Folate Intake	1134	
Low		378 (33.33)
Middle		378 (33.33
High		378 (33.33)
Proportion of Low usual Folate intake	1134	378 (33.3)

Values are mean ± SD, median [IQR], or frequency (percent)

^#^Mean ± (SD)

*age in Months

^a^Median (25th; 75th percentile)

^b^Protestant, Jehovah-witness

^c^sister, daughter, aunt

^d^unskilled and manual labor, clerical

^e^280 observations

Many of the participants reported using their food production as a primary source of food and travel more than half a kilometer for reaching the source. The median dietary diversity score was 4.0 (IQR: 3.0; 5.0) and 35.4% of had optimum dietary diversity (consumed 5 or more food groups daily). Most study participants had under-five children in their household with a median age of 36 months (IQR: 23.0; 48.0). The highest number of previous pregnancies reported was thirteen (Table 1).

### Food frequency distribution and food ranking

**Fig. 1:**
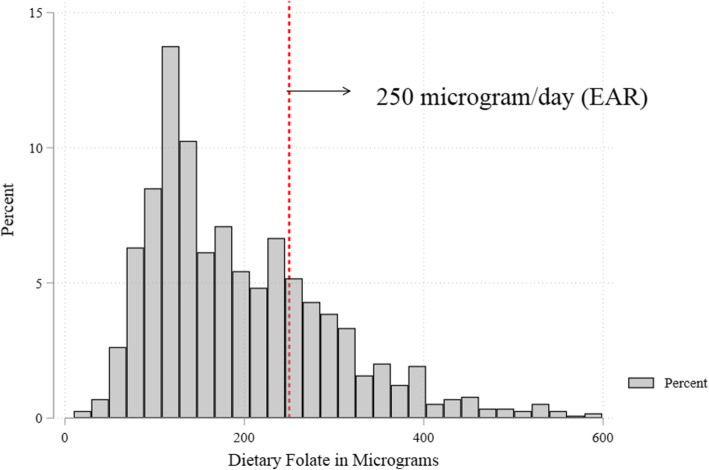
Usual dietary Folate consumption among women of reproductive age, Kersa, Eastern Ethiopia, 2019

**Fig. 2:**
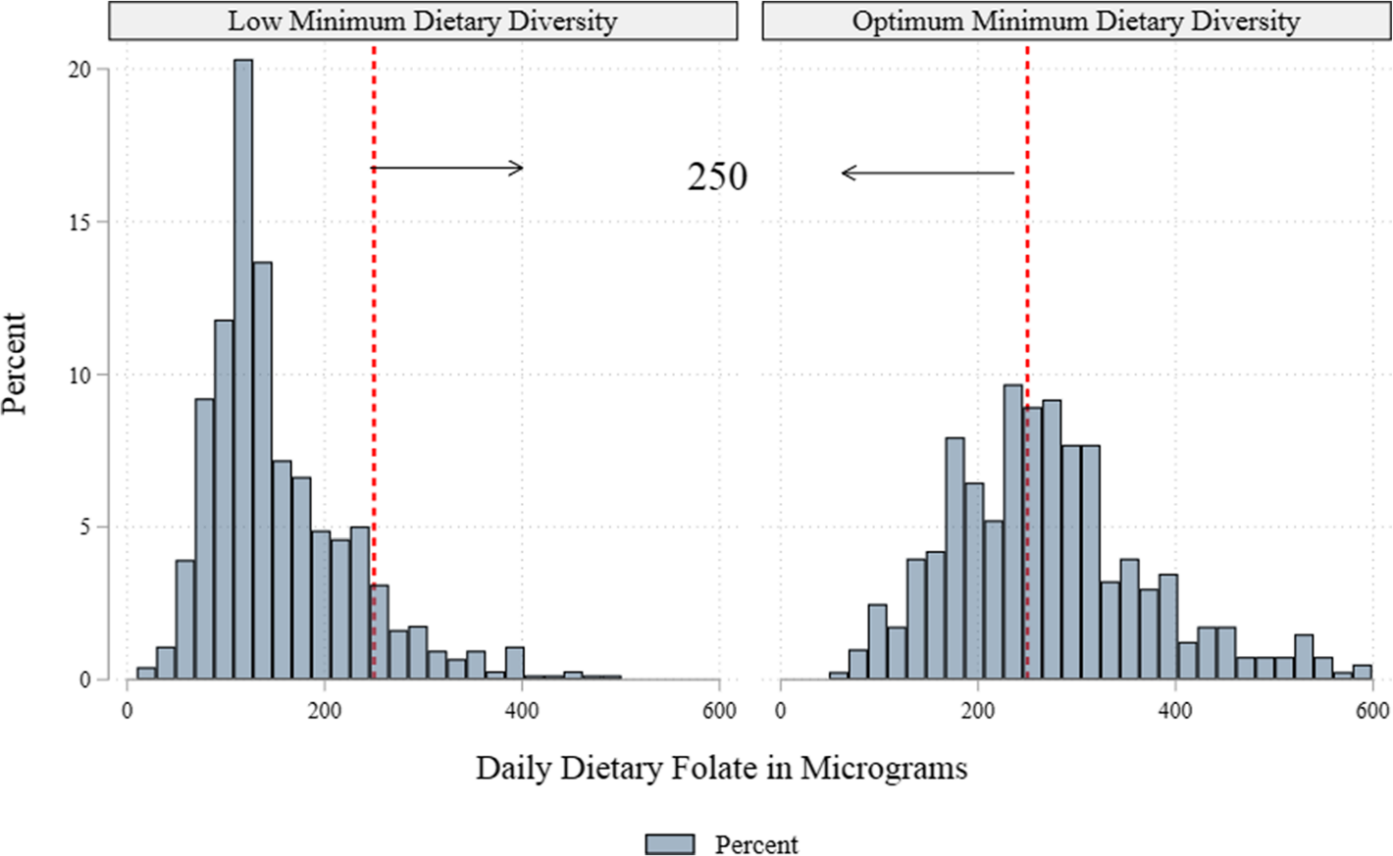
Total Dietary Folate Consumption by Minimum Dietary Diversity among women of reproductive age, Kersa, Eastern Ethiopia, 2019

Table 2 shows the ranking and contribution of food groups to the dietary intake of folate. Almost all participants reported consuming starchy staples and other vegetables but these groups ranked 2nd and 5th in contributing to daily dietary folate intake. Even though less than half of the study participants reported intake of beans and peas, they were ranked the 1st in contributing dietary folate with a median of 101.7 μg/d (IQR: 73.7; 178.3). The least consumed food group was flesh foods and it is also contributed least to folate intake. The median folate consumption in this study was 170.2 μg/day (IQR: 118.3; 252.2): 95% CI (164.3–176.1). The distribution of folate intake was positively skewed and 73.9% were at risk for dietary folate inadequacy based on a cut-off of 250 μg/day (Figs. 1 and 2). About 33% of WRA had low folate intake.

**Table 2:** Food frequency with Mean Folate dietary intake of women of reproductive age in Kersa, Eastern Ethiopia, 2019

Food group	Rank	Contribution	Consumed	Folate (ug/d) ^a^
1) All starchy staples	2	49.6%	1130 (99.6)	84.4 (60.4; 104.0)
Consumed everyday			769 (67.8)	95.2 (75.9; 110.4)
Consumed ≤6 days			361 (31.8)	55.1 (38.6; 72.4)
2) Beans and Peas	1	59.7%	467 (41.2)	101.7 (73.7; 178.3)
Consumed everyday			16 (1.4)	340.0 (129.0; 342.3)
Consumed ≤6 days			451 (39.8)	97.1 (73.7; 161.6)
3) Nuts and Seeds	4	11.0%	74 (6.5)	18.7 (18.0; 27.0)
Consumed everyday			0.0 (0.0)	0.0 (0.0; 0.0)
Consumed ≤6 days			74 (6.5)	18.7 (18.0; 27.0)
4) All Diary	8	4.4%	754 (66.5)	7.5 (4.3; 7.5)
Consumed everyday			293 (25.8)	7.5 (7.5; 7.5)
Consumed ≤6 days			461 (40.6)	5.4 (4.3; 6.9)
5) Flesh Foods	10	1.0%	119 (10.5)	1.7 (0.9; 3.4)
Consumed everyday			4 (0.4)	6.0 (6.0; 148.0)
Consumed ≤6 days			115 (10.1)	1.7 (0.9; 3.4)
6) Eggs	9	3.1%	134 (11.8)	5.3 (2.6; 10.6)
Consumed everyday			2 (0.2)	18.5 (18.5; 18.5)
Consumed ≤6 days			132 (11.6)	5.3 (2.6; 10.6)
7) Vitamin A-rich dark green leafy vegetables	3	23.0%	524 (46.2)	39.2 (26.1; 52.3)
Consumed everyday			10 (0.9)	91.4 (91.4; 91.4)
Consumed ≤6 days			514 (45.3)	39.2 (26.1; 52.3)
8) Other vitamin A-rich vegetables and fruits	6	5.5%	355 (31.3)	9.4 (6.0; 16.9
Consumed everyday			2 (0.2)	21.0 (21.0; 21.0)
Consumed ≤6 day			353 (31.1)	9.0 (6.0; 16.3)
9) Other vegetables	5	10.4%	1113 (98.2)	17.7 (15.5; 27.1)
Consumed everyday			890 (78.5)	17.7 (15.5; 28.0)
Consumed ≤6 days			223 (19.7)	15.5 (11.9; 23.5)
10) Other fruits	7	5.2%	152 (13.4)	8.8 (8.8; 23.1)
Consumed everyday			20 (1.8)	42.8 (42.8; 42.8)
Consumed ≤6 days			132 (11.6)	8.8 (8.8; 13.2)

^a^Median Folate intake (25th; 75th percentile)

### Factors associated with dietary folate inadequacy

Table 3 shows the factors associated with inadequate dietary folate consumption. We found that wealth index, seasonal employment, and low women’s nutritional diversity were associated with inadequate folate intake defined as intake of below EAR of folate, which is below 250 μg/day in a population in univariate models. In adjusted models, seasonal employment, food source, being in the lowest and middle wealth index category, and low women’s nutritional diversity were associated with dietary folate inadequacy. Women with low dietary diversity intake were twice as likely (APR 1.9; 95% CI 1.7–2.2) to have inadequate folate intake compared to women who met the criteria for minimum dietary diversity. Women who were involved in seasonal agricultural employments were more likely (APR 1.1; 95% CI 1.1–1.2) to have inadequate folate intake compared to women with full-time employment. Compared to women in the wealthiest households, women from poor and middle wealth tertile were 1.1 times (95% CI 1.0–1.3) and 1.2 times (95% CI 1.1–1.4) more likely to have dietary folate inadequacy, respectively. Women aged 15–25 years were 10% less likely to be at risk for folate inadequacy compared to those aged 36 years or older.

**Table 3:** Factors associated with inadequate dietary folate consumption^a^ among women of reproductive age in Kersa, eastern Ethiopia, 2019

	Inadequate Folate N (%)	CPR	95% CI	*P*-value	APR	95% CI	*P*-value
Age							
16–25	190 (70.9)	0.88	0.80–0.97	0.01	0.89	0.80–0.97	0.01**
25–35	464 (72.4)	0.91	0.84–0.98	0.02	0.96	0.89–1.02	0.20
≥ 36	184 (80.4)	ref			ref		
Employment type							
Full-time	547 (71.5)	ref			ref		
Part-time	62 (61.4)	0.86	0.73–1.01	0.3	0.93	0.80–.1.07	0.30
Seasonal	229 (85.5)	1.19	1.12–1.28	0.000	1.12	1.06–1.20	< 0.001**
BMI							
Underweight	149 (79.3)	1.08	0.99–1.17	0.40	1.03	0.96–1.11	0.4
Normal	640 (73.6)	ref	ref		ref	ref	
Overweight	49 (64.5)	0.88	0.74–1.04	0.58	0.97	0.84–1.11	0.6
Wealth Index							
Poor	513 (80.5)	1.39	1.24–1.57	0.01	1.14	1.03–1.26	0.01**
Middle	191 (72.1)	1.25	1.09–1.43	0.000	1.20	1.06–1.35	< 0.001**
Rich	134 (57.8)	ref	ref		ref	ref	
Number of previous pregnancies ^#^	4.54 (2.44)	1.02	1.00–1.03	0.03	0.99	0.98–1.01	0.7
Dietary Diversity							
Optimum	180 (44.9)	ref	ref		ref	ref	
Low	658 (89.8)	2.00	1.79–2.23	0.000	1.94	1.73–2.18	< 0.001**
Food source							
Household	622 (72.8)	ref	ref		ref	ref	
Market	216 (77.1)	1.06	0.98–1.14	0.2	1.07	1.01–1.15	0.04**

^a^folate intake of < 250 μg/day

^#^ mean (SD)

** significant at *p* = 0.05

*CPR* Crude Prevalence Ratio, *APR* Adjusted Prevalence Ratio

## Discussion

This study assessed dietary folate intake among women of reproductive age in Kersa, Eastern Ethiopia. The food groups least consumed were fish, eggs, fleshy foods, and fruits. The majority of women had folate intake which was insufficient and far less than the recommended standard of 250 μg/d [32]. We found that women that had low dietary diversity, in poorer households, seasonal employment, and market purchases of food were at higher risk of dietary folate inadequacy. Older women were also more like to have inadequate dietary folate intake.

The magnitude of the folate inadequacy in this study was higher compared to Tanzania which was 33.8%, but comparable with the low intake of folate, which was 33% [35]. It was also higher than in the Nigerian study, where 47% had inadequate intake but lower than in the South African report of 98% [6]. The difference could be related to the difference in utilizing different methodologies, food stability, and security in those different countries.

The high magnitude of dietary folate inadequacy is expected and could be related to the characteristics of the study area. With reliance on supplementation of folic acid in pregnancy, WRA would be at risk for folate deficiency. It is also one of drought-prone, with poor living standards and difficulty in accessing affordable folate-rich foods and poor place in Ethiopia. Most of the dietary system is mainly based on traditional farming in unsuitable places, with poor support from the agriculture system. As a result, most of the residents are supported through the safety-net program [36].

In contrast, the developed counties had decreased folate deficiency and the incidence of NTD by fortifying primary foods that would typically have no or little folate [9, 35, 37] In those countries not only mandatory folate fortification policies are in place, but also improving in dietary diversity, gender equity and equality [4, 38, 39] unlike Ethiopia, explaining the higher folate deficiency in our population. It is estimated that mandatory fortification in Ethiopia will reduce NTD by 85% annually if fully implemented [40]. Although effective, the policy has not been endorsed and developed in Ethiopia [41].

We found that with an increase in women’s age was more likely to have inadequacy of dietary folate. Another cross-sectional FFQ study reported younger women were more likely to have folate inadequacy than advanced-aged women [7]. This difference could be respectively be explained by the higher household family member and children the older women expected to feed [7]. The study finding could also be limited by the potential introduction of recall bias and participants could over-report the consumption of specific food items.

Seasonal agricultural employment and being in a poor and middle category of wealth were also associated with dietary folate intake insufficiency. This finding is expected because women’s seasonal dependent agricultural employment could have a potential for hunger and food scarcity and insecurity for families due to lack of other options if difficulties arise for harvest or drought seasons [42]. Besides, seasonal agricultural employment also leads to poverty, which in-turn poses makes it difficult to purchase adequate nutrient-rich food for the family [43]. The risk of folate dietary inadequacy increased twice in women who had low dietary diversity compared to their counterparts. This finding can be attributed to the fact that having low dietary diversity leads to unhealthy and unbalanced diet patterns as well as micronutrient deficiencies [44]. WRA in Ethiopia relatively eat less because of food shortage, physical discomfort, and unpleasant monotonous food with less variety [45]. This puts them at increased risk for any micronutrient deficiency in a household. Other studies in Ethiopia have also reported dietary diversity was a strong predictor of micronutrient adequacies with a direct relationship with food security, household income, and health access of a community [46, 47].

Ethiopia is one the highest NTD burdened country, with a prevalence rate ranging from 0.23–40.3% [48, 49]. For pregnant women, reports indicate 12% folate deficiency in Ethiopia, 3% in Kenya, and 4% in Nigeria [6]. Low levels of folate consumption reported in this study can affect nutrition and health for WRA. Given that low folate intake can affect cell growth and duplication [50]. Low intake among WRA prior to and during pregnancy could lead to irreversible damage to the nerve system of the conceived fetus [51]. The nerve damage to the baby ranges from a complete loss of fetal brain to some defects in the brain, spinal cord, and associated structures [52]. In any case of these, the outcome is clear, either the fetus will die or be born with permanent neural damage leading to a lifelong disability affecting growth, development, and failure to thrive [53].

To correct the problem, in the routine health system, pregnant women are given a capsule that contains iron and folic acid for ninety days. Yet, it is reported that only as few as 5 % of the women complete the full doses, and the remaining more than 95% leave their fetus to the mercy of dietary folate consumption [54, 55]. In addition, the widely available foods in Ethiopia have low bioavailable folate. Even though it is planned in introducing Folic Acid intervention program in our national document like a fortification, it is not implemented [27].

Some of the strength of this study is utilizing the first community-based FFQ with adequate sample size and training data collectors for quality control. The utilization of FFQ is a quick and efficient way of identifying and assessing micronutrient inadequacy. A past-week FFQ can provide a better assessment of the usual intake of micronutrient intake compared to 24- h recall [56]. However, it has also several limitations. FFQ usually overestimates micronutrient intake which made it difficult to accurately capturing absolute micronutrient value. Also, the serum level of folate and serving size of the food intake was not measured, which introduces with and between variation errors [57]. To reduce this we have seen the folate intake distribution using two different cut-offs, the EAR(< 250 μg/d) and tertiles. Other factors that may affect folate absorption, seasonal dietary changes, knowledge, and awareness towards folate were not considered in this study.

## Conclusion

The study found that folate intake is low and that folate inadequacy is a major public health problem in Kersa, Eastern Oromia. Diversifying diets, and daily consumption of folate-rich foods like beans and liver, and mandatory fortification of wheat or salt are highly recommended to increase folate adequacy and decrease the risk of folate deficiency among WRA. National FFQ coupled with plasma folate levels is recommended for accurate identification of folate inadequacy and deficiency as well as for monitoring of micronutrient deficiencies.

## Data Availability

The datasets used and analyzed during this study are available from the corresponding author on reasonable request.

## Abbreviations

DDS: Dietary diversity score
FFQ: Food frequency questionnaire
IFA: Iron and folic acid
MDD: Minimum dietary diversity
NTD: Neural tube defect
PCA: Principal component analysis
SSA: Sub-Saharan Africa
WRA: Women of reproductive age
